# Enamel Remineralization Competence of a Novel Fluoride-Incorporated Bioactive Glass Toothpaste—A Surface Micro-Hardness, Profilometric, and Micro-Computed Tomographic Analysis

**DOI:** 10.3390/tomography7040063

**Published:** 2021-11-09

**Authors:** Imran Farooq, Saqib Ali, Faraz Ahmed Farooqi, Jehan AlHumaid, Mashael Binhasan, Sara Shabib, Fahim Vohra, Tariq Abduljabbar

**Affiliations:** 1 Faculty of Dentistry, University of Toronto, Toronto, ON M5G 1G6, Canada; 2 Department of Biomedical Dental Sciences, College of Dentistry, Imam Abdulrahman Bin Faisal University, Dammam 31441, Saudi Arabia; drsaqiibali@gmail.com; 3 Department of Dental Education, College of Dentistry, Imam Abdulrahman Bin Faisal University, Dammam 31441, Saudi Arabia; fafarooqi@iau.edu.sa; 4 Department of Preventive Dental Sciences, College of Dentistry, Imam Abdulrahman Bin Faisal University, Dammam 31441, Saudi Arabia; jaalhumaid@iau.edu.sa; 5 Department of Restorative Dentistry, Division of Operative Dentistry, Collage of dentistry, King Saud University, Riyadh 60169, Saudi Arabia; mbinhasan@ksu.edu.sa (M.B.); sashabib@ksu.edu.sa (S.S.); 6 Department of Prosthetic Dental Science, College of Dentistry, King Saud University, Riyadh 11545, Saudi Arabia; fvohra@ksu.edu.sa (F.V.); tajabbar@ksu.edu.sa (T.A.)

**Keywords:** dental enamel, bioactive glass, fluoride, toothpaste, surface micro-hardness, surface roughness, micro-CT

## Abstract

This study aimed to analyze the enamel remineralization efficacy of a novel fluoridated bioactive glass (F-BG) toothpaste compared to a standard fluoride toothpaste. Seventy-two enamel blocks (N = 72) were divided into groups of twenty-four blocks according to the toothpaste exposure—group 1: brushed with distilled water, group 2: brushed with fluoride toothpaste (Colgate^TM^), and group 3: brushed with F-BG toothpaste (BioMinF^TM^). Pre-brushing, enamel blocks were demineralized using 6 wt.% citric acid (pH = 2.4). Tooth brushing was performed using a mixture of respective toothpaste and artificial saliva (AS), and each enamel block received 5000 linear strokes. The samples were assessed for surface micro-hardness (to estimate Vickers hardness number, VHN), surface roughness (Ra), and volume loss/gain using micro-computed tomography (micro-CT). The highest increase in the VHN was noticed for group 3 (117.81) followed by group 2 (61.13), and all the intragroup comparisons were statistically significant (*p* < 0.05). Demineralization increased the Ra values, and a decrease was observed post-remineralization for all the groups. The maximum Ra decrease was observed for group 3 (−223.2 nm) followed by group 2 (−55.6 nm), and all the intragroup comparisons were again statistically significant (*p* < 0.05). Micro-CT investigation revealed that the enamel volume decreased after demineralization and increased after remineralization among all groups. The F-BG toothpaste showed greater enamel surface micro-hardness (increased VHN), smoother surface (low roughness), and better volume restoration (remineralization) in comparison to the fluoride toothpaste.

## 1. Introduction

Dental caries is an infectious multifactorial disease that is prevalent worldwide [1], and it can affect 60 to 90% of the global population [2]. An imbalance between the processes of demineralization and remineralization causes dental caries that ultimately results in the loss of tooth structure [3]. Many factors can augment the process of demineralization, and examples include the consumption of sugary snacks (that are easily metabolized by cariogenic bacteria to produce acids), eating high acidic foods, and intake of fizzy drinks with low pH [4]. Dental public health stakeholders have been working tirelessly over the past few decades to come forward with cost-effective strategies that are beneficial in decreasing the incidence of dental caries [5]. To reduce the demineralization and cavitation of tooth surfaces, the most effective strategy accepted to date is brushing the teeth with a toothpaste that contains a remineralizing active ingredient [6]. One such active ingredient commonly used inside toothpaste is fluoride, which reduces the incidence of dental caries and favors the remineralization process [7]. It is a well-documented fact that fluoride content and its available concentration in toothpaste have a direct relation with dental caries prevalence [8]. Fluoride can replace hydroxyl (OH^−^) ions in the apatite structure and form fluorapatite (FAP), which is more stable and acid-resistant [9], therefore resulting in a decline in dental caries incidence and the reversal of existing carious lesions [10]. Additionally, fluoride ions can inhibit microbial growth and metabolism, leading to less acid production and reduced demineralization [11].

Bioactive glass (BG) is another remineralizing material that has gained popularity in dentistry in the last decade and can be used for various clinical dental applications [12]. Any material capable of forming a hydroxyl-carbonated apatite (HCA)-like layer inside a biological environment is called a bioactive material [13]. BG (which is composed of calcium sodium phosphosilicate) is such a material that immediately interacts with cells and tissues to form an HCA-like layer inside an aqueous solution [14], such as saliva. These glasses were introduced initially for bone regeneration purposes, but due to the close resemblance between the chemical composition of bone and enamel structure, their use has increased in dentistry [15]. Most noticeably, they are currently being used as remineralizing agents inside toothpaste and as a coating for dental implants [16]. The original BG composition, called 45S5 or Bioglass^TM^, contained 45 wt.% silica, 24.5 wt.% sodium oxide, 24.5 wt.% calcium oxide, and 6 wt.% phosphorus pentaoxide [17]. Since its introduction in the late 1960s, various modifications have been made in the composition of BGs to tailor fit them for several clinical applications. Of late, a novel BG that contains fluoride inside the glass composition was introduced [18]. This fluoridated-BG (F-BG) toothpaste contains 5% fluorocalcium phosphosilicate and entails the ability to form FAP rapidly [19] after exposure to saliva. Human saliva neutralizes the acidic pH in the oral cavity and plays a major role in remineralization. Compositionally, the presence of calcium, phosphate, and fluoride ions facilitates the penetration of these remineralizing ions into the demineralized portions of enamel [20]. A toothpaste containing all these ions could therefore, potentially improve the cariostatic potential of saliva.

A major portion of dental research is directed towards the development and testing of novel dentifrices that have optimum remineralizing properties. This research could lead to the prevention of dental caries, and in turn, the needless removal of tooth structure during restorative procedures can be avoided. In a previous study, it was demonstrated that enamel remineralization potential could be enhanced if two remineralizing agents are used in combination due to their synergistic effect [21]. F-BG toothpaste contains fluoride that is incorporated in its BG composition; hence, it would be interesting to investigate its enamel remineralization competence. The comparative data on fluoride and F-BG remineralizing toothpaste concerning enamel surface loss, topography, and hardness are not available in indexed literature. Therefore, the present study aimed to assess the relative influence of F-BG toothpaste with standard fluoride toothpaste on enamel remineralization using surface micro-hardness (Vickers hardness number—VHN), volume assessment, and the reduction in enamel’s surface roughness. It was hypothesized that brushing enamel surfaces with F-BG toothpaste would lead to an improvement in their surface micro-hardness and volume and reduce their surface roughness.

## 2. Materials and Methods

Ethical approval was attained from the institution’s ethics review committee. All the protocols were stringently shadowed in line with the recommendations of the ethics committee and in accordance with the Helsinki Declaration of 1975 and its further modifications.

### 2.1. Preparation of Enamel Blocks

Seventy-two extracted maxillary first premolar teeth were collected from the institution’s Oral Surgery clinics. Teeth that were devoid of any apparent deformities, restorations, cracks, plaque, and calculus deposits were only included in our study. The teeth were cut just over the cemento-enamel junction by means of a water-cooled diamond saw (Isomet^TM^ 5000 Linear Precision Saw, Buehler Ltd., Lake Bluff, IL, USA) with a blade speed of 2500 rpm, and a 10 mm/min feed rate was utilized. The roots of the sectioned teeth were discarded, and the anatomical crowns were retained. The crowns of the teeth were then used to prepare enamel blocks by embedding them in self-cure acrylic (Ivolen, Ivoclar Vivadent, Liechtenstein, Germany) blocks of~5 *×* 5 mm^2^ with labial surfaces exposed. The visible enamel surfaces were ground and polished (1200 grit size) inside a grinding and polishing machine (MetaServ^TM^ 250 Grinder-Polisher with Vector Head, Buehler Ltd., Lake Bluff, IL, USA). All the enamel blocks were stored in artificial saliva (AS) before, during, and after experiments.

### 2.2. AS Preparation

The AS was synthesized in line with the formula recommended earlier by Fusayama et al. [22]. Briefly, 0.4 g of NaCl and KCl, 0.69 g of NaH_2_PO_4_.H_2_O, 0.795 g of CaCl_2_.H_2_O, and 0.005 g of Na_2_S.9H_2_O were added incrementally and mixed thoroughly in 1 L of deionized water. The pH of freshly synthesized AS was mildly acidic (pH = 5.3), which was moved up to a neutral pH of 7 by the addition of aliquots of 1 M NaOH.

### 2.3. Demineralization Procedure

In order to induce artificial demineralization in the teeth, all samples were exposed to 400 mL of 6 wt.% citric acid (pH = 2.4) for 5 min. To depict in vivo demineralization, the enamel blocks were demineralized inside citric acid-containing glass beakers that were placed on top of an oscillating orbital shaker (CO-Z^®^ Orbital Shaker, Lenaxa, KS, USA). Post-demineralization with citric acid, the teeth were cleaned with distilled water for 1 min and dried inside a desiccator (Dry-Keeper^TM^ Desiccator Cabinets, Cole-Parmer^TM^, Leicestershire, UK) before further investigation.

### 2.4. Grouping of Enamel Blocks

The enamel blocks (N = 72) were randomly and equally divided into three parts so that for each investigational technique (surface micro-hardness, surface roughness, and micro-CT), twenty-four teeth are assessed. The grouping was performed on the basis of the toothpaste that was used for simulated brushing (remineralization)—group 1: enamel blocks were brushed with distilled water, group 2: enamel blocks were brushed with fluoride toothpaste (Colgate^TM^, Colgate-Palmolive Arabia Ltd., Dammam, Saudi Arabia), and group 3: enamel blocks were brushed with BG toothpaste (BioMinF^TM^, BioMin Technologies Ltd., London, UK).

### 2.5. Simulated Tooth Brushing Protocol

Toothpaste slurries were used for all the groups to perform simulated tooth brushing (except group 1, where distilled water was used). The slurries were made by mixing the toothpaste with AS in a 1:2 ratio, and tooth brushing was accomplished inside a simulated brushing machine (Toothbrush simulator; model ZM-3.8, Feldkirchen-Westerham, Germany) with soft-bristled toothbrushes (Oral B^®^, Pro-Flex^TM^, California, CA, USA). The load applied was 200 gm, and each enamel block received 5000 linear strokes (not exceeding 150 strokes/min). The respective slurries were replenished every 4 min (600 brushing strokes). After the completion of the brushing cycle, distilled water was used to wash the samples, which were then returned to their individual containers with AS.

### 2.6. Surface Micro-Hardness Testing

The surface micro-hardness testing for the enamel blocks was performed at three stages: baseline, post-demineralization, and post-remineralization. The Vickers hardness number (VHN) was calculated with the help of a digital micro-hardness tester (FM-ARS 9000; Future-Tech Corp, Kawasaki, Japan). Each enamel block received three indentations at each stage, and an average hardness value was noticed. The load applied was 100 gm, and the dwell time was 10 s.

### 2.7. Surface Roughness Analysis

Surface roughness analysis was again performed at three stages (similar to micro-hardness analysis). A non-contact profilometer (Contour GT Optical Microscopes profilometer, Bruker, Tucson, AZ, USA) was utilized in our study for analysis. Each enamel block was scanned three times, and an average roughness value (Ra) was noticed. The Ra represents mean height of the peaks and depth of valleys in the measuring length from a mean line. All the scans were performed inside the distinct area on the enamel surface.

### 2.8. Micro-CT Investigation

To observe volumetric and surface changes in the surface of enamel that can be caused by demineralization or remineralization, micro-CT was performed inside the scanner (SkyScan 1172, version 1.5; Bruker Micro-CT, Kontich, Belgium). The samples were scanned at three time points (baseline, post-demineralization, and post-remineralization), similar to surface micro-hardness and roughness analysis. The enamel volume of each sample was calculated, and then the mean volume for the whole group (group 1, group 2, and group 3) was estimated for each time point. This was then followed by the comparison between groups (inter-group comparison). The enamel volume was also compared within the group for different time points (intra-group comparison). To scan the samples, the source voltage used was 100-kV, source current was 100-μA, and image pixel size was 100-μA. Additionally, an Al + Cu filter was applied, and the image format was TIFF with 1600-msec exposure. Additional parameters included rotation step of 0.700°, frame averaging of 3, random movement of 10, and rotation of 360°. These raw images were recreated using the NRecon software (Bruker SkyScan, Aartselaar, Belgium). For the calculation of enamel volume (mm^3^), the exact same area of all the samples was scanned using the CTscan software (Bruker SkyScan, Aartselaar, Belgium).

### 2.9. Statistical Analysis

The data were gathered in Microsoft Excel 365, then exported to SPSS (Statistical Package for Social Science, version 22, Inc., Chicago, IL, USA) for analysis. Descriptive statistics were presented as Mean ± Standard Deviation (SD). Wilcoxon Signed rank test was applied to evaluate intragroup comparisons (baseline vs. post-demineralization and baseline vs. post remineralization). The Kruskal–Wallis test was used to assess the intergroup differences. A *p*-value of less than 0.05 was considered statistically significant.

## 3. Results

### 3.1. Surface Micro-Hardness Outcomes

**Figure 1:**
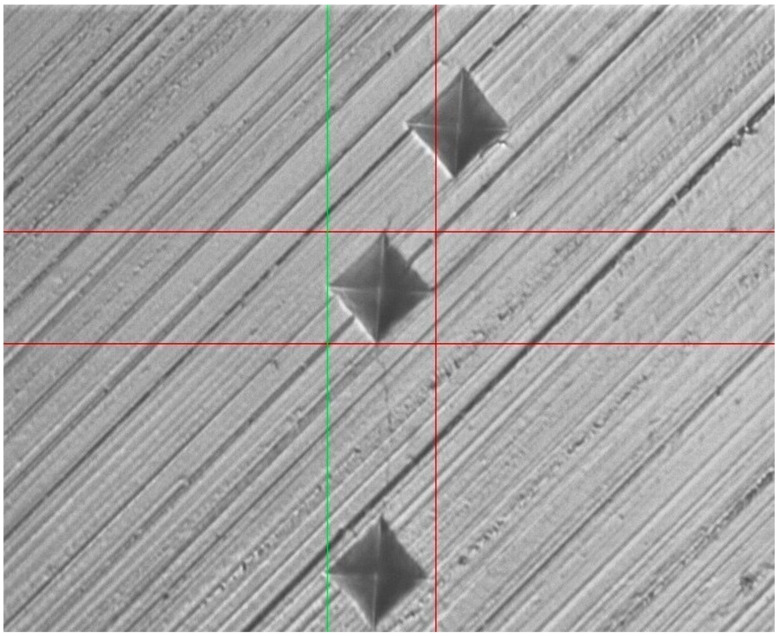
Vickers indentation executed to assess the micro-hardness of enamel surfaces.

Surface micro-hardness analysis was carried out to assess the VHN of enamel blocks belonging to different groups. Figure 1 represents Vickers indentation executed to assess the micro-hardness of enamel surfaces. The results of surface micro-hardness analysis revealed a decrease in the hardness value of all the groups after demineralization and an increase post-remineralization. Although none of the groups were able to restore the VHN to the baseline, the highest increase in mean VHN after remineralization was noticed for group 3 (F-BG toothpaste) (117.81), followed by group 2 (fluoride toothpaste) (61.13) (Table 1). All the intragroup comparisons were statistically significant (*p* < 0.05), and on the intergroup comparison, the only statistically significant difference was found at the baseline stage (Table 1).

### 3.2. Surface Roughness Outcomes

**Figure 2:**
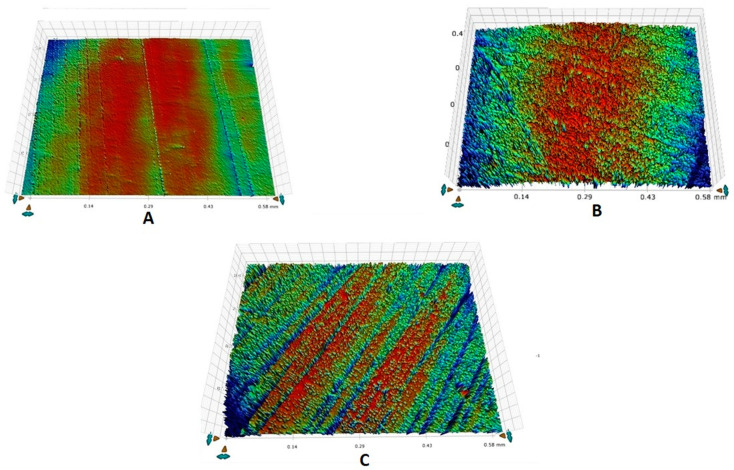
Representative enamel surface roughness profilometric images belonging to group 1 showing: (**A**) ground and polished enamel surface at baseline, (**B**) rough enamel surface post-demineralization, (**C**) remineralized enamel surface after brushing.

**Figure 3:**
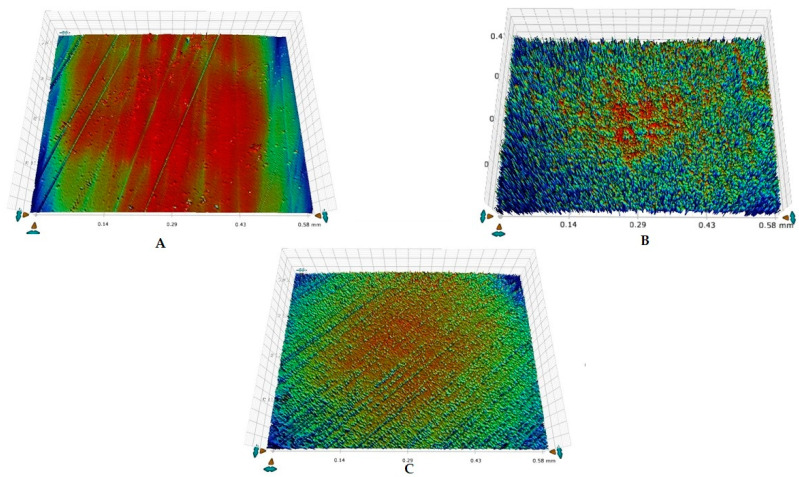
Representative enamel surface roughness profilometric images belonging to group 2 showing: (**A**) ground and polished enamel surface at baseline, (**B**) rough enamel surface post-demineralization, (**C**) remineralized enamel surface after brushing.

**Figure 4:**
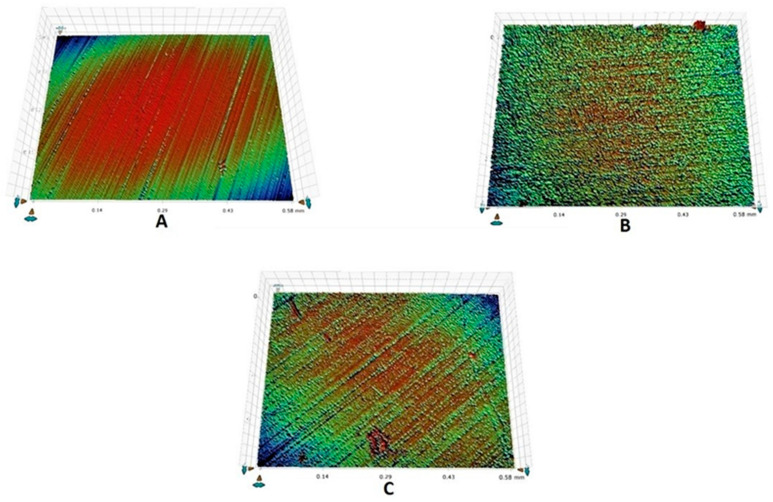
Representative enamel surface roughness profilometric images belonging to group 3 showing: (**A**) ground and polished enamel surface at baseline, (**B**) rough enamel surface post-demineralization, (**C**) remineralized enamel surface after brushing.

It was observed that demineralization caused an increase in the mean Ra for all the groups and a decrease in Ra was observed post-remineralization. All the intragroup comparisons were statistically significant (*p* < 0.05), whereas all the intergroup comparisons were not significant (*p* > 0.05) (Table 2). The highest decrease in mean Ra was observed for group 3 (−223.2), followed by group 2 (−55.6). The representative enamel surface profiles for groups 1, 2, and 3 taken at different time intervals are shown in Figure 2A–C, Figure 3A–C, and Figure 4A–C, respectively.

### 3.3. Micro-CT Outcomes

**Figure 5:**
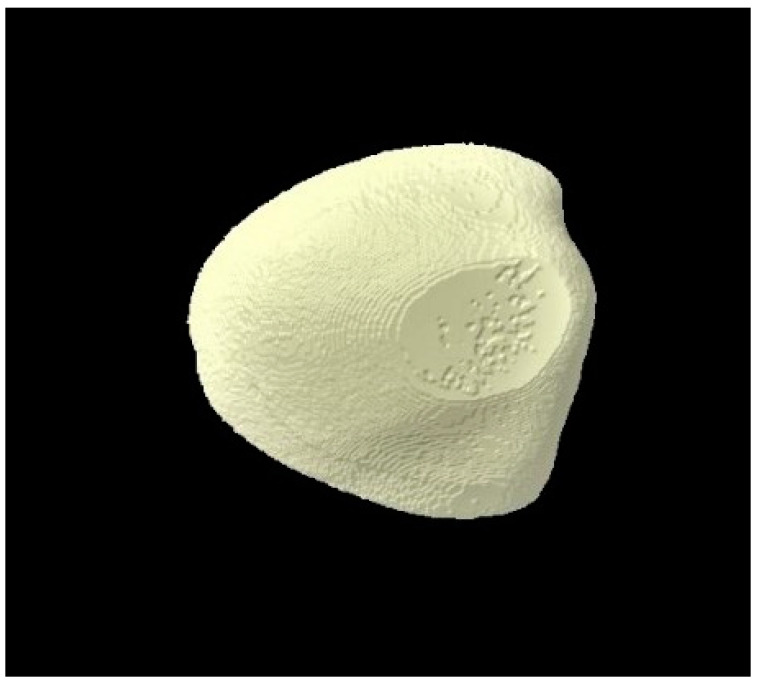
Micro-CT image of group 2 samples presenting enamel surface at baseline.

**Figure 6:**
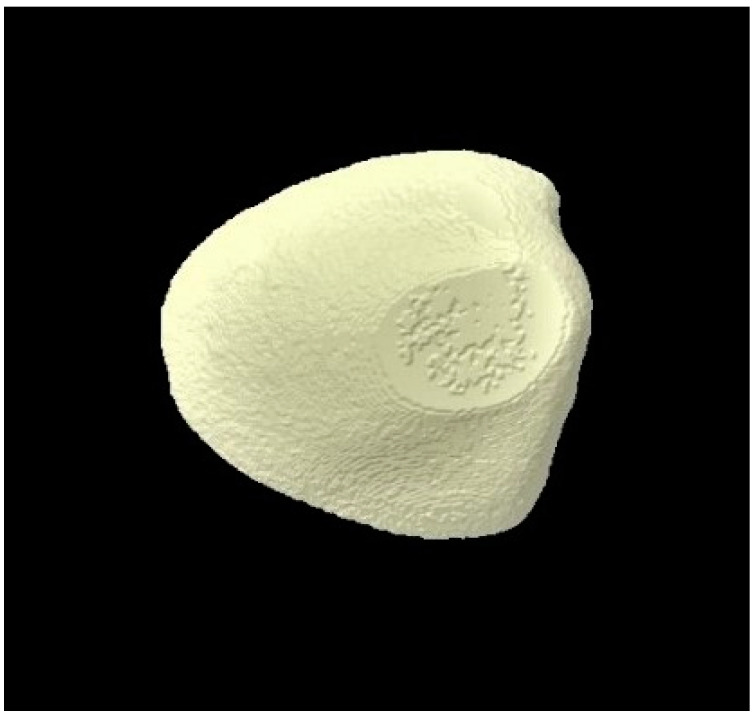
Micro-CT image of group 2 samples presenting enamel surface post-demineralization.

**Figure 7:**
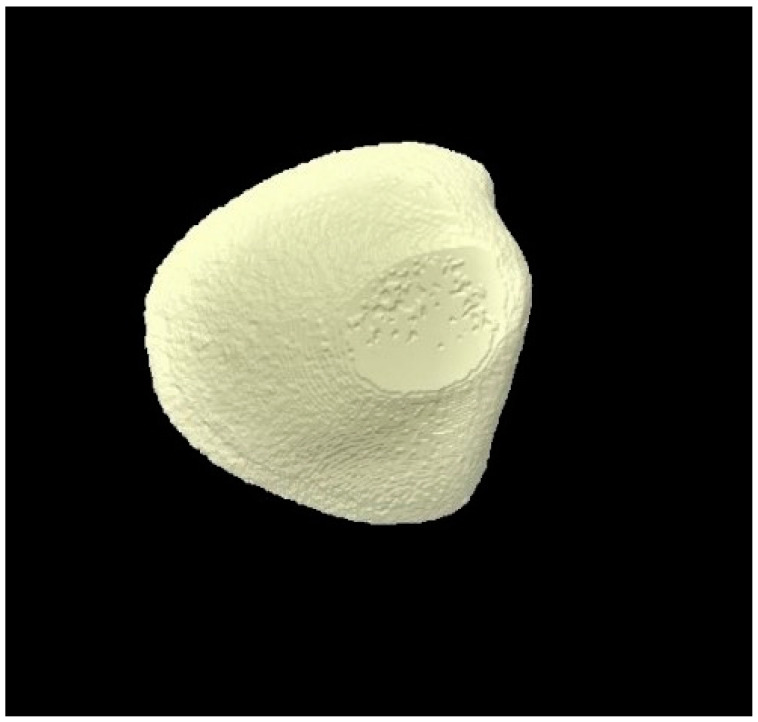
Micro-CT image of group 2 samples presenting enamel surface post-remineralization.

**Figure 8:**
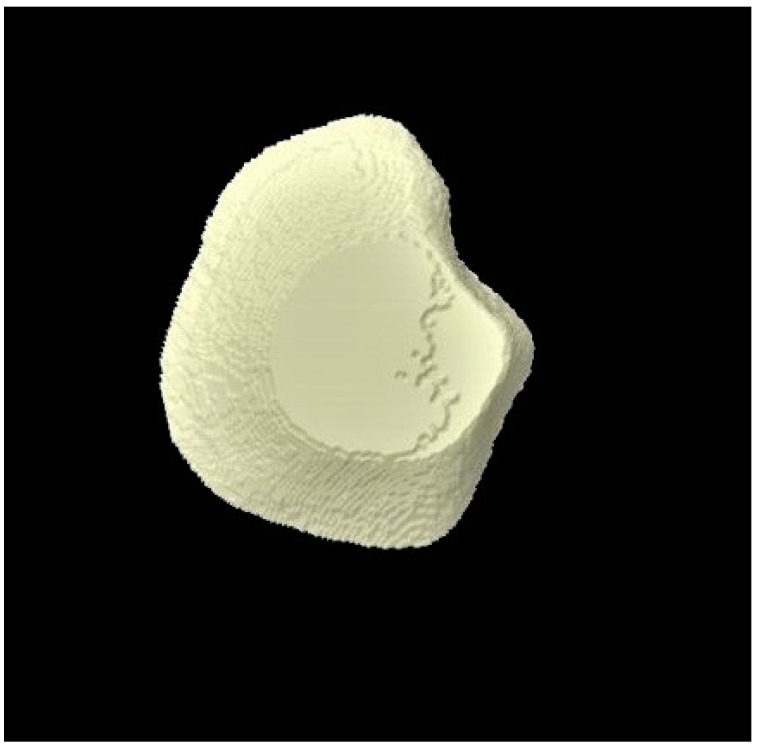
Micro-CT image of group 3 samples presenting enamel surface at baseline.

**Figure 9:**
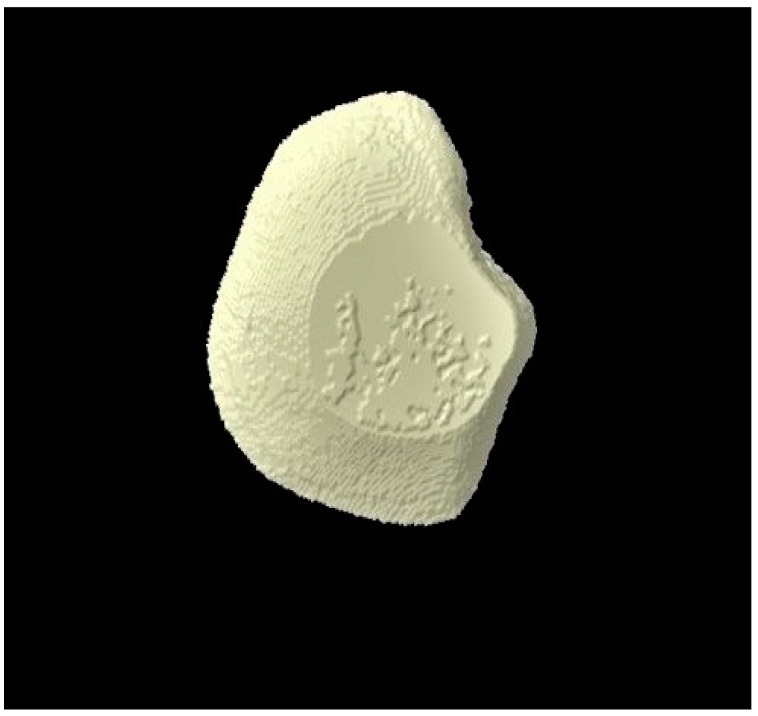
Micro-CT image of group 3 samples presenting enamel surface post-demineralization.

**Figure 10:**
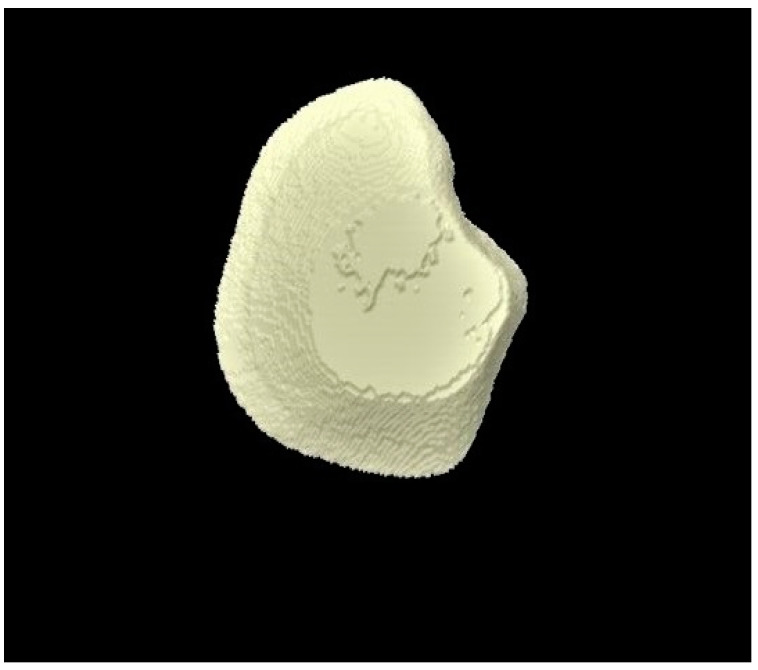
Micro-CT image of group 3 samples presenting enamel surface post-remineralization.

The micro-CT investigation results demonstrated that for each group, enamel volume decreased after demineralization and increased after remineralization (Table 3). The mean enamel volume ± SD for all the groups decreased after demineralization and increased after remineralization. The representative micro-CT images for group 1 are not shown as they were similar, and no visual change was observed. The representative micro-CT images for group 2 taken at different time intervals are shown in Figure 5 (baseline), Figure 6 (post-demineralization), and Figure 7 (post-remineralization). The representative micro-CT images for group 3 taken at different time intervals are shown in Figure 8 (baseline), Figure 9 (post-demineralization), and Figure 10 (post-remineralization). Since the differences were slight, none of the intergroup and intragroup comparisons were statistically significant (*p* > 0.05).

## 4. Discussion

The findings of our study have revealed that F-BG toothpaste performed better than standard fluoride toothpaste and distilled water in terms of increasing enamel’s surface micro-hardness and volume and reducing its roughness. Based on this study’s results, the hypothesis that the use of F-BG toothpaste on enamel’s surface would increase its micro-hardness and volume and reduce surface roughness was accepted. However, it should be noted that although the increase in enamel volume was observed more for our F-BG toothpaste group, the differences were non-significant, and this aspect may require further evaluation in future studies.

Surface micro-hardness testing using Vickers indentations was used in our study to evaluate the hardness of enamel surfaces. This method has been deemed appropriate to measure surface hardness levels with high precision [23]. One major advantage of this technique includes the possibility of repeated measurements with the application of different loads within a limited time [24]. Considering these benefits, we chose Vickers surface micro-hardness testing to be the technique to assess the hardness levels of our samples. Previously, Srivastava et al. reported that F-BG toothpaste performed better than Novamin^TM^ and casein-phosphopeptide amorphous calcium phosphate (CPP-ACP) with fluoride in their study and resulted in significantly greater remineralization of demineralized enamel lesions [25]. Similar results were conveyed by another in vitro study that was conducted by Mohapatra et al., who reported that brushing with F-BG toothpaste improved the Vickers hardness of the enamel surface post-demineralization, and this improvement was greater as compared with Novamin^TM^ [26]. Another study by Ali et al. reported similar findings and revealed that F-BG toothpaste contains more soluble fluoride than Novamin^TM^ and also resulted in higher micro-hardness levels as compared with its counterpart [27]. Our results are in agreement with these studies, although we compared F-BG toothpaste with a standard fluoride toothpaste instead of Novamin^TM^. A probable reason for F-BG toothpaste to show better remineralization potential could be the fact that fluoride deposited on the tooth surface via a normal toothpaste is washed away quickly by salivary flow [27]. Therefore, the amount of FAP formed due to the limited bioavailability of fluoride is questionable. In F-BG toothpaste, fluoride is incorporated inside its glass composition, which possibly leads to its sustained release along with other useful ions such as calcium and phosphate [28,29], leading to the formation of FAP and a consequent increase in surface micro-hardness. Another aspect of our surface-microhardness results was the observance of reduced enamel micro-hardness post-demineralization for all the samples and an increase in the micro-hardness post-remineralization. The literature shows that it is a common finding to observe a reduction in the hardness values of human enamel samples after demineralization and an increase in the hardness values post-remineralization [30], and our findings are in agreement with the literature in this aspect.

Another aspect of our study analyzed the reduction in surface roughness of enamel blocks post-brushing. The enamel surface becomes rough after an acidic challenge and smooths after remineralization [31]. Surface roughness or non-contact profilometry is a suitable method to assess surface changes in enamel and has been used by various researchers in the past [31,32,33]. Studies that have assessed enamel surface roughness after the application of our tested F-BG toothpaste are scarce in the literature. In an earlier study by Bakry et al., it was demonstrated that extracted human premolar teeth showed a decrease in surface roughness when these were brushed with BG toothpaste [34]. Another study verified the reduction in enamel’s surface roughness values after the samples were immersed in a mixture of F-BG toothpaste and AS [35]. Our findings are in conformity with these studies as the most significant reduction in the roughness of enamel surfaces post-brushing was achieved by BG toothpaste-brushed samples. F-BG toothpaste contains a glass with high phosphate content [19], and in a previous study, Brauer et al. established that high phosphate concentration in the glass helps it to maintain its network connectivity, thus ensuring the development of a FAP layer [36] that could reduce roughness. Another important feature of F-BG toothpaste that could play an essential part in reducing surface roughness could be its small particle size. In an earlier study, Tie et al. compared the particle size of F-BG toothpaste with Novamin^TM^ and concluded that the former has an average particle size of 5.92 μm, which is much smaller than the average particle size of the latter (14.47 μm) [37]. The smaller particle size could make the toothpaste less abrasive (making it less gritty), thereby reducing enamel surface roughness. Although this aspect was not investigated in our study, it could be useful in the decline in surface roughness of enamel surfaces brushed with BG toothpaste, as observed in our study.

We also utilized micro-CT to assess changes in the volume of enamel surfaces at different stages of the experiment. Micro-CT is a non-destructive method of investigating enamel surface changes that provides three-dimensional (3D) high-definition images and has been used by various researchers to study volumetric changes in enamel [38,39]. In an earlier micro-CT study, Farooq et al. reported a significant increase in enamel surface volume when they were immersed in a mixture of F-BG toothpaste and AS as compared with Novamin^TM^ and AS mixture [35]. Our study has presented similar results, and F-BG toothpaste-brushed samples presented with more restored enamel volume than the fluoride toothpaste and distilled water group. The restoration of enamel volume post-brushing could be linked again to the release of remineralizing ions such as calcium, phosphate, and fluoride from F-BG toothpaste that result in the formation of FAP instead of HCA. Fluoride-containing BGs can selectively dissolute in an aqueous solution and release bioactive ions [40]. After the release of ions, apatite is formed, which precipitates on the enamel surface, followed by biological fixation that prevents further dissolution, thus preserving the biological structure [41]. Tooth brushing with a fluoride toothpaste results in elevated fluoride levels immediately; however, they are decreased incrementally and are back to baseline levels after about 2 h [42]. This is not the case with F-BGs as the level of fluoride remains elevated even after 2 h as compared with the baseline levels [28]. These elevated levels of fluoride could result in enhanced remineralization, as observed by enamel volumetric changes observed for F-BG toothpaste-brushed samples in our study. It should be noted here that F-BG toothpaste contains a low concentration of fluoride incorporated in its glass composition (530 ppm) [26], as compared with the fluoride toothpaste used in our study (label claim: 1450 ppm). This low concentration not only helps in the prevention of undesirable fluorite formation [29], but also ensures the long-term delivery of fluoride ions for the formation of a stable FAP layer [35].

Periodontitis is a common inflammatory disease that results in the destruction of periodontal tissues [43]. The use of BG-based particulates can not only repair periodontal osseous defects [44], but they are also effective in the subgingival debridement of periodontal pockets when used as an air-polishing powder [45]. Recently, F-BG was incorporated in dental composites. It was observed that F-BG composites exhibited comparable strengths to the existing composites after immersion in AS after 84 days [46]. As the strength of dental resin composite is questionable when compared to dental amalgam [47], the inclusion of F-BG resulting in its improved strength [46] is a good sign and paves the way for further research in this area that could potentially lead towards the development of dental resin composites with superior aesthetics and strength.

One of the limitations of our study was its in vitro nature. The real in vivo environment is dynamic and could present multiple challenges. Another limitation is the recording of hardness and roughness values at different stages from the same exposed area on the enamel surface. However, to counter this, we marked the area on which experiments were going to be performed before the testing, and every effort was made to perform analysis in the same area. The micro-CT technique also entails some limitations. Helical and multisection technique artifacts can be produced during the image reconstruction process that could have resulted in modifying enamel volume slightly in our study. The reader should be cautious and should keep all these limitations in mind while interpreting our results.

## 5. Conclusions

The F-BG toothpaste, in comparison to fluoride toothpaste, showed greater surface micro-hardness (VHN), a smoother enamel surface (low surface roughness), and better enamel volume restoration (remineralization) within the limitations of the experiment. Future in vitro studies and in vivo trials validating the formation of FAP and clinical remineralization potential of F-BG toothpaste are recommended.

## Figures and Tables

**Table 1:** Showing mean ± SD VHN for the three groups at different analysis time points.

Surface Micro-Hardness Analysis Time Points	Group 1 (Distilled Water)	Group 2 (Fluoride Toothpaste)	Group 3 (BG Toothpaste)	*p*-Values *
Baseline	572.11 ± 28.38 ^+^	480.75 ± 66.04 ^+^	502.46 ± 74.5	0.017
Post-demineralization	123.87 ± 54.60 ^a^	211.28 ± 150.09 ^a^	167.71 ± 170.8 ^a^	0.471
Post-remineralization	173.13 ± 73.94 ^a^	272.13 ± 137.01 ^a^	285.52 ± 161.18 ^a^	0.27
*p*-values **	0.0001	0.003	0.003	
Difference (post-remin–post-demin)	49.26	61.13	117.81	

* *p*-values among the groups, ** *p*-values within the groups, ^a^ significant differences on intragroup comparison, ^+^ significant differences on intergroup comparison, VHN = Vickers hardness number.

**Table 2:** Showing mean ± SD Ra (nm) for the three groups at different analysis time points.

Surface Roughness Analysis Time Points	Group 1 (Distilled Water)	Group 2 (Fluoride Toothpaste)	Group 3 (BG Toothpaste)	*p*-Values *
Baseline	249.2 ± 127.0	276.2 ± 190.0	221.4 ± 106.3	0.925
Post-demineralization	1148.3 ± 816.8 ^a^	1114.1 ± 506.1 ^a^	992.0 ± 479.2 ^a^	0.912
Post-remineralization	1105.8 ± 825.9 ^a^	1058.5 ± 492.5 ^a^	768.8 ± 300.8 ^a^	0.547
*p*-values **	0.02	0.001	0.0001	
Difference (post-remin–post-demin)	−42.2	−55.6	−223.2	

* *p*-values among the groups, ** *p*-values within the groups, ^a^ significant differences on intragroup comparison.

**Table 3:** Showing mean ± SD enamel volume (mm^3^) as shown by micro-CT for the three groups at different analysis time points.

Micro-CT Analysis Time Points	Group 1 (Distilled Water)	Group 2 (Fluoride Toothpaste)	Group 3 (BG Toothpaste)	*p*-Values *
Baseline	15.58 ± 6.38	14.72 ± 2.35	17.24 ± 10.39	0.968
Post-demineralization	15.02 ± 6.31	14.54 ± 2.25	16.82 ± 10.33	0.983
Post-remineralization	15.03 ± 6.39	14.55 ± 2.26	17.01 ± 10.40	0.949
*p*-values **	0.779	0.835	0.852	
Difference (post-remin–post-demin)	0.01	0.01	0.19	

* *p*-values among the groups, ** *p*-values within the groups.
